# A mechanism review of metal phthalocyanines as single-atomic catalysts in electrochemical energy conversion

**DOI:** 10.1039/d5sc03210e

**Published:** 2025-07-14

**Authors:** Zikang Li, Ziqi Zhou, Mingzi Sun, Tong Wu, Qiuyang Lu, Lu Lu, Baian Chen, Cheuk Hei Chan, Hon Ho Wong, Bolong Huang

**Affiliations:** a Department of Chemistry, City University of Hong Kong Tat Chee Avenue, Kowloon 999077 Hong Kong SAR b.h@cityu.edu.hk

## Abstract

Metal phthalocyanines (MPcs) are emerging as model single-atom catalysts (SACs) with atomically defined MN_4_ cores and tailorable peripheries, enabling precise mechanistic explorations and rational performance tuning. Herein, we review recent progress on carbon-supported MPc catalysts for key electrochemical energy-conversion reactions, including the oxygen reduction/evolution (ORR/OER), hydrogen evolution (HER), CO_2_ reduction (CO_2_RR) and nitrogen/nitrate reduction (N_2_RR/NO_*x*_RR) reactions. We emphasize mechanistic insights obtained from density-functional theory (DFT) and how π–π stacking, defect engineering, and curvature in graphene or carbon nanotubes modulate the electronic structure of MPcs, optimize intermediate adsorption, and suppress competing pathways. Meanwhile, we focus on specific computational methods like grand-canonical DFT (GC-DFT) and *ab initio* molecular dynamics (AIMD), which provide potential- and solvent-explicit descriptions of reaction energetics, bridging gaps between conventional constant-charge calculations and experimental observations. Besides, the machine-learning (ML) applications in MPc screening and identification based on metal centers, axial ligands, and dual-site motifs are discussed, followed by a future outlook of remaining challenges and further development of next-generation MPc-based catalysts for sustainable energy technologies.

## Abstract 2

This review discusses metal phthalocyanine-based single-atom catalysts for electrochemical energy conversions, emphasizing reaction mechanisms and computational methods for mechanism exploration and catalyst screening.

## Graphical abstract

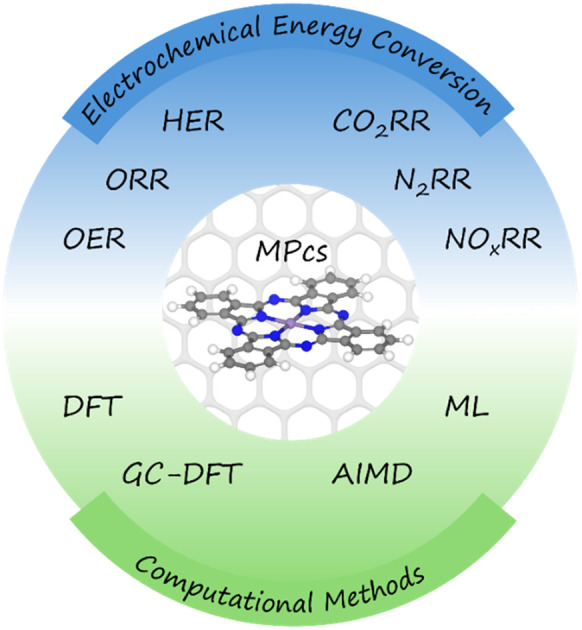

## 1. Introduction

Fossil fuels, which currently dominate the energy sector, not only contribute to greenhouse gas emissions but also face finite availability. The environmental issues brought about by the overuse of fossil fuels, like climate change, air pollution, and ecosystem degradation, have increased global attention to address sustainable energy development. To this end, electrochemical technologies, such as water-splitting electrolyzers, CO_2_ reduction reactors, and fuel cells, have emerged as promising solutions for converting sustainable energy sources into storable chemical fuels like hydrogen or hydrocarbons. These systems bridge the gap between intermittent renewable energy generation and consistent industrial demand, positioning clean energy conversion as a cornerstone of both environmental sustainability and economic resilience.

A wide range of catalysts have been developed for clean energy conversion, including metal-based catalysts,^1^ single-atom catalysts (SACs),^2^ carbon-based metal-free catalysts,^3^*etc.* Among them, metal phthalocyanines (MPcs) as some of the M–N–C type SACs have attracted increasing research interest due to their well-defined molecular structures and good catalytic activities.^4–14^ Compared to normal M–N–C type SACs, in spite of disadvantages such as low dispersion of metal active sites that leads to lower activity, MPcs offer the advantages of straightforward and scalable synthesis and diverse ligand derivatives that can be prepared for various applications, and their precise structural composition facilitates a deeper understanding of reaction mechanisms at the molecular level and enables thorough kinetic evaluations of catalytic performance.^15^ Moreover, MPcs have been used for different types of catalysts based on their inherent properties and structure modifications, such as acting as photosensitizers in photocatalysts,^16^ forming porous organic polymers *via* polymerization,^17^ building metal organic frameworks (MOFs)^18,19^ or covalent organic frameworks (COFs)^20,21^ and pyrolyzing into carbon-based SACs.^22–24^ These modifications improve catalytic efficiency and enable precise control over product selectivity in energy conversion.

To date, MPcs have been applied in various electrocatalytic energy conversions, which have been discussed regarding the characteristics, performances, and developments of FePc, CoPc, and NiPc in energy conversions, and have elucidated structure–performance relationships.^25–31^ However, plenty of the experimental studies on electrocatalysts rely on traditional trial-and-error approaches. The conventional methods, whether through experimental synthesis and evaluation or numerical simulations, are heavily dependent on the expertise and intuition of researchers, often leading to suboptimal results. The inherent limitations of this approach hinder the improvements in catalytic performance both in time and space. Besides, traditional research methodologies cannot efficiently manage the complexity of systems involving numerous variables with a heavy workload.^32^ In the era of rapid advancements in data science and computational technology, there is an urgent need for more effective, data-driven strategies to accelerate the discovery of novel electrocatalyst candidates and identify optimal material combinations, ultimately enhancing efficiency and innovation in this labor-intensive field.^33–35^

**Fig. 1:**
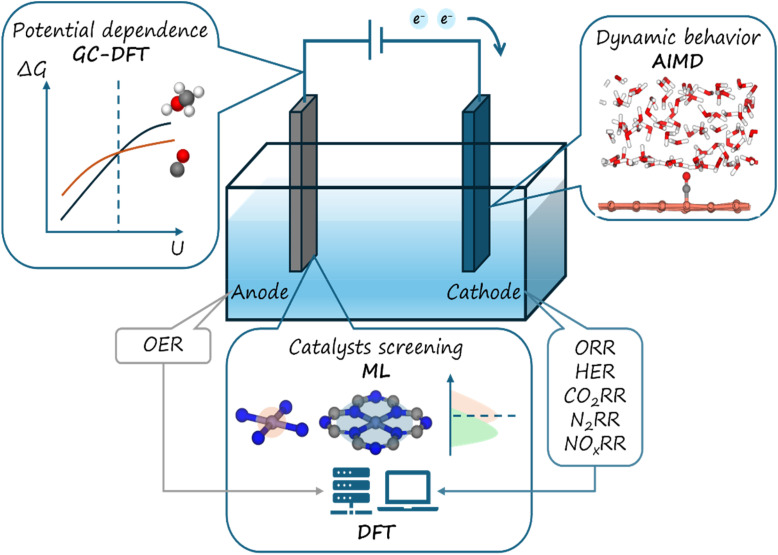
An overview of MPc electrochemical energy conversions and applied computational methods in this review.

In this review, we provide a comprehensive overview of recent advancements in carbon-supported metal phthalocyanine (MPc) catalysts for energy conversion (Fig. 1). Specifically, we focus on research conducted using density functional theory (DFT) calculations, along with other computational methods that offer deeper insights into electrocatalytic systems. First, we summarize the catalytic mechanisms of MPc catalysts that are used in various electrochemical reactions, including the hydrogen evolution reaction (HER), oxygen evolution reaction (OER), oxygen reduction reaction (ORR), carbon dioxide reduction reaction (CO_2_RR) and nitrogen/nitrite/nitrate reduction reaction (N_2_RR/NO_*x*_RR). Given the critical role of carbon-based substrates in MPc catalysts, we also explore the structure–activity relationship between the molecular catalyst and its substrate, as revealed by theoretical studies. Furthermore, we introduce computational methods, such as grand-canonical DFT (GC-DFT) and *ab initio* molecular dynamics (AIMD) simulations, which aim to simulate a more explicit representation of the electrocatalytic environment and reveal the details of the mechanism. Additionally, we discuss the integration of machine learning (ML) approaches for molecular screening and theoretical guidance in MPc catalyst design. Finally, we outline the key challenges and future perspectives for MPc-based catalysts in this rapidly evolving field. This review highlights the significance of theoretical studies in advancing MPc-based catalysis and lays the groundwork for structural engineering in SACs for energy conversion applications.

## 2. Electrochemical energy conversions of MPcs

### 2.1 ORR/OER/HER

Hydrogen and oxygen related energy conversion reactions have been studied for decades, and are the fundamental reactions of water electrolysis and fuel cells.^36,37^ The mechanisms for these reactions are well investigated in different possible pathways as shown in Table 1, based on acidic or alkaline media in the reduction system.

**Table 1:** Reaction formulae of the ORR/OER/HER in different mechanisms

Acidic media	Alkaline media
**ORR**	
* + O_2_ → *O_2_	
*O_2_ + H^+^ + e^−^ → *OOH	*O_2_ + H_2_O + e^−^ → *OOH + OH^−^
*OOH + H^+^ + e^−^ → *O + H_2_O	*OOH + e^−^ → *O + OH^−^
*O + H^+^ + e^−^ → *OH	*O + H_2_O + e^−^ → *OH + OH^−^
*OH + H^+^ + e^−^ → * + H_2_O	*OH + e^−^ → * + OH^−^

**OER**	
* + H_2_O → *OH + H^+^ + e^−^	* + OH^−^ → *OH + e^−^
*OH → *O + H^+^ + e^−^	*OH + OH^−^ → *O + H_2_O + e^−^
*O + H_2_O → *OOH + H^+^ + e^−^	*O + OH^−^ → *OOH + e^−^
*OOH → * + O_2_ + H^+^ + e^−^	*OOH + OH^−^ → * + O_2_ + H_2_O + e^−^
	
**HER**	
* + H^+^ + e^−^ → *H	* + H_2_O + e^−^ → *H + OH^−^
*H + H^+^ + e^−^ → * + H_2_	*H + H_2_O + e^−^ → * + H_2_ + OH^−^
2 *H → 2 * + H_2_	

The ORR can be categorized into two main pathways based on the number of electrons transferred: the four-electron and two-electron pathways.^38,39^ In the four-electron pathway, molecular oxygen (O_2_) is directly reduced to either water (H_2_O) in acidic media (O_2_ + 4H^+^ + 4e^−^ → 2H_2_O) or hydroxide ions (OH^−^) in alkaline media (O_2_ + 2H_2_O + 4e^−^ → 4OH^−^), without the formation of H_2_O_2_ and can follow either dissociative or associative mechanisms, depending on the oxygen dissociation barrier on the adsorbing atom. In contrast, the two-electron pathway leads to the formation of hydrogen peroxide (H_2_O_2_), which affects the fuel cell applications due to its lower efficiency and potential side reactions. For MPc SACs, the associative mechanism is favorable since the O–O bond can be activated through metal coordination.

The OER is the reverse reaction of the ORR that requires a strong water affinity on the adsorbing atom.^40^ Owing to the high selectivity on the single reaction site, SACs prefer an additional step on the *OOH intermediate rather than generating dual *O for oxygen association. Like the HER, it involves two transferred electrons with two possible mechanisms: the Volmer–Heyrovsky and Volmer–Tafel process.^41,42^ Unlike the ORR and OER, the easy H binding on the surrounding atoms of the reaction site may offer an additional adsorbed H atom for the Tafel step, which can be attributed to the neighboring effect on the SACs.

**Fig. 2:**
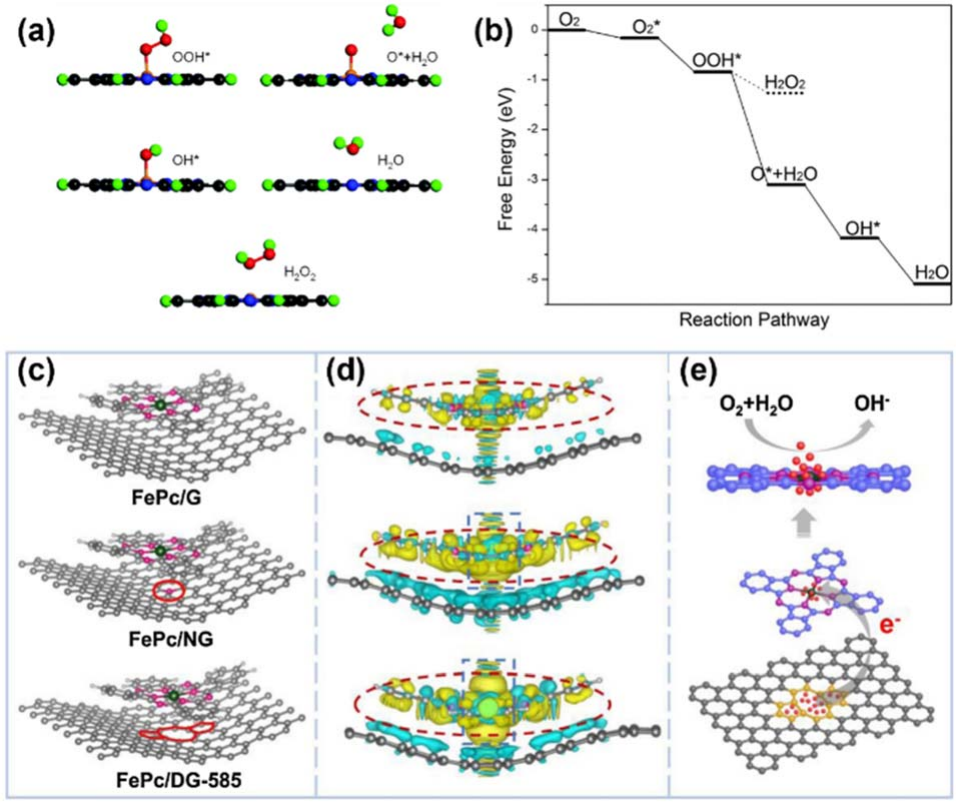
(a) The optimized structures of intermediates (OOH*, O*, OH*, H_2_O, and H_2_O_2_) along the reaction path of the ORR proceeded on the FePc monolayer. (b) Free energy profile for the ORR at zero potential. The solid and dotted lines denote the 4e and 2e reduction pathways, respectively.^44^ (c) The top views of optimized FePc/G, FePc/NG and FePc/DG-585 based hybrid interfaces. (d) The side views of the charge density difference plot for the interfaces between G, NG and the DG-585 sheet and the FePc layer. Yellow and cyan isosurfaces represent charge accumulation and depletion. (e) The schematic diagram of the probable ORR electrocatalytic mechanism of FePc/DG-585. The red spheres represent electrons.^7^ Reproduced from ref. 44 and 7 with permission from Royal Society Chemistry and Elsevier, copyright 2015, 2021.

Early in 2013, Jiang *et al.* prepared the graphene-supported FePc (g-FePc) composite with good ORR activity in alkaline media, matching that of the commercial Pt/C catalyst.^43^ In 2015, Wang *et al.* demonstrated that the two-dimensional FePc monolayer exhibits high catalytic activity for the ORR in acidic environments, which is another non-platinum alternative for fuel cells.^44^ They applied GGA with the PBE functional and found that O_2_ molecules adsorb on the Fe center in an end-on configuration, activating the O–O bond *via* charge transfer and elongation, which facilitates efficient reduction (Fig. 2(a) and (b)). This demonstration was examined in 2016 when Wang *et al.* synthesized an FePc polymer sheathed on multiwalled carbon nanotubes (MWCNTs).^45^ These research studies on MPcs integrated with carbon materials have revealed that the carbon substrates are pivotal in creating a robust, conductive, and durable structure that maximizes the ORR activity of FePc-based SACs. Yang *et al.* studied FePc with single-walled carbon nanotubes (SWCNTs) and found that when FePc is anchored on SWCNTs, the π–π stacking between the planar FePc molecules and the curved graphitic surface of SWCNTs induces a rehybridization of Fe 3d orbitals, modifying the electronic state of the Fe center.^46^ This interaction increases the electron density around the Fe atom, facilitating stronger adsorption of O_2_ molecules and weakening the O–O bond in intermediates like OOH, thereby promoting a more efficient 4-electron ORR pathway. Besides, Yu *et al.* conducted a DFT modulation on FePc with defective graphene in ORR applications.^7^ Compared to normal graphene (G) and nitrogen-doped graphene (NG), they found that the defective graphene with specific 585 topology defects (DG-585) significantly enhances ORR performance of FePc through tailored electronic interactions, specifically charge redistribution, transferring electrons to Fe atoms in FePc, and creating an electron-rich environment (Fig. 2(c–e)). This electron transfer upshifts the d-band center of Fe atoms, strengthening O_2_ adsorption and accelerating reaction kinetics.

**Fig. 3:**
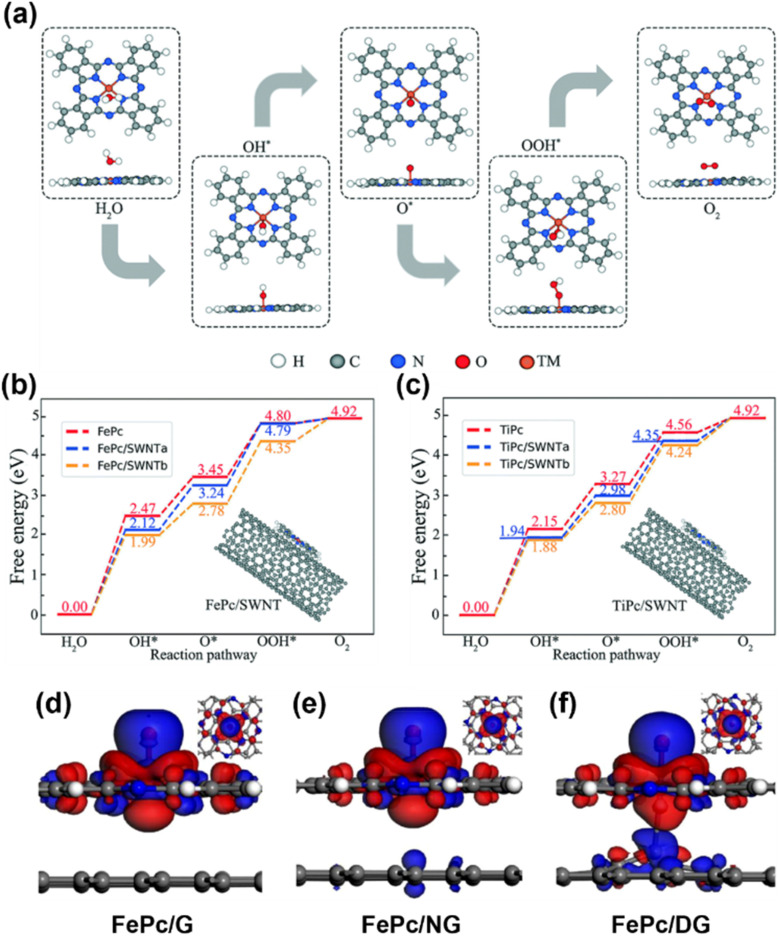
(a) The optimized structures of OER reactants and intermediates (H_2_O, OH*, O*, OOH*, and O_2_) on the MPc. (b) and (c) The OER pathway of FePc and TiPc with SWNT supports, respectively.^50^ (d)–(f) The charge density difference plots of the adsorbed O atom on FePc. The charge accumulations (blue color) occur mainly around the adsorbed O atom and the depletion (red color) is centered on the Fe atom. FePc is supported on pure graphene (d), nitrogen doped graphene (e), and defective graphene (f).^51^ Reproduced from ref. 50 and 51 with permission from Royal Society Chemistry and Elsevier, copyright 2020, 2022.

The first research on MPc-based SACs for the OER was proposed by Ladouceur *et al.*, who have pyrolyzed CoPc with carbon black and conducted the OER in acidic environments.^47^ Based on this, Li and his colleagues reported a sulfonated CoPc/CNT hybrid that had both ORR and OER performance.^48^ Aralekallu *et al.* studied CoPc polymer-coated Ni foam as an OER catalyst and they examined the catalytic enhancement from the metal phthalocyanine by mixing it with the benchmark catalyst IrO_2_.^49^ Nevertheless, the applications of MPcs in the OER are still limited by energy barriers. To overcome these challenges, researchers proposed some methods to enhance the OER activity of MPcs by studying the substrate effect in the catalyst system. Wan and his colleagues demonstrated that the OER performance of 3d-block transition metal MPcs can be further enhanced by the charge transfer between the catalysts and SWCNTs.^50^ The carbon substrates reduced the OER energy barrier for almost every step, which is attributed to the weaker interaction between the O atom and H atom of adsorbed water molecules on MPc/SWCNTs than on individual MPcs (Fig. 3(a–c)). Zhang *et al.* conducted a first-principles investigation to explore the influence of graphene-based substrates on the OER performance of FePc.^51^ They demonstrated an analogous substrate-driven effect in OER catalysis to those observed in ORR catalysis: the integration of a graphene substrate enhances electron transfer between FePc and oxygen-containing intermediates. Their findings showed that FePc displays varying OER activity depending on the graphene substrate type, with defective graphene exhibiting a significant enhancement in catalytic performance (Fig. 3(d–f)). This improvement was attributed to axial interactions between the iron center in FePc and carbon atoms in the defective graphene substrate, which optimized the adsorption strength of oxygen-containing intermediates, thereby balancing catalytic efficiency and stability.

In addition, the HER is typically considered a competing reaction to other reduction processes such as the CO_2_RR and N_2_RR, making it less attractive for energy conversion applications. However, Kwon *et al.* discovered that FePc/MoS_2_ two-dimensional composites exhibited enhanced HER catalytic activities, in which FePc promoted the reaction on the surface of MoS_2_ through electron concentration according to the partial density of states (PDOS) analysis.^52^

### 2.2 CO_2_RR

Converting CO_2_ into energy-rich molecules presents a dual-benefit strategy that mitigates atmospheric CO_2_ levels while generating industrially valuable products. For instance, CO_2_ can be reduced to single-carbon (C_1_) products like CO, formic acid (HCOOH), formaldehyde (CH_2_O), methanol (CH_3_OH), or methane (CH_4_), depending on the electrode material and applied potential. Researchers have explored diverse methods for this purpose, including thermal,^53^ chemical^54^ and photochemical techniques.^55–58^ However, these approaches face limitations such as high operational costs, suboptimal product selectivity, inefficient energy use, scalability challenges, and stringent reaction conditions. In comparison, the electrocatalytic CO_2_RR has become a potential solution due to its good selectivity and efficiency in synthesizing C_1_ or multi-carbon (C_2+_) products, and adaptability to industrial applications.^31,59^ Despite these advantages, the CO_2_RR faces two critical challenges: first, sluggish reaction kinetics caused by the stability of CO_2_ molecules; and second, competition from the HER at the electrode–electrolyte interface, which reduces the overall process efficiency.^60,61^

The catalytic activity of MPcs in CO_2_ reduction was first reported by Meshitsuka *et al.* in 1974.^62^ Their pioneering work involved compositing various phthalocyanines with graphite as reaction electrodes, revealing that nickel phthalocyanine (NiPc) and cobalt phthalocyanine (CoPc) exhibited promising CO_2_ reduction performance. In recent years, significant advancements have been achieved in single-atom catalysts based on metal phthalocyanines for CO_2_ reduction. A methylated nickel phthalocyanine-multiwalled carbon nanotube (NiPc-MWCNTs) composite demonstrated high activity in converting CO_2_ to CO, achieving 300 mA cm^−2^ with a faradaic efficiency exceeding 99.5%.^63^ Similarly, a cobalt phthalocyanine-multiwalled carbon nanotube (CoPc-MWCNTs) composite enabled CH_3_OH synthesis, attaining a faradaic efficiency of 44% at high overpotentials.^64^ These breakthroughs highlight the versatility of metal phthalocyanine-based structures in tailoring catalytic pathways for CO_2_ conversion into value-added products.

The mechanisms governing the CO_2_RR on MPcs are primarily described through two pathways: the sequential proton-electron transfer (SPET) mechanism and the proton-coupled electron transfer (PCET) mechanism.^65–67^ These frameworks explain how CO_2_ molecules adsorb onto catalytic sites and undergo subsequent intermediate transformations. In the two-electron CO_2_RR process, which generates CO and HCOOH, product selectivity is critically influenced by the electronic structure of the active metal center on the catalyst.^68–70^ This structural property governs both the reaction energy barriers and the adsorption strength of intermediates. For example, copper phthalocyanine (CuPc) uniquely facilitates HCOOH production in 3d transition metal-based Pc,^71^ while most MPcs preferentially catalyze CO formation due to their distinct electronic configurations that favor specific intermediate stabilization.

**Fig. 4:**
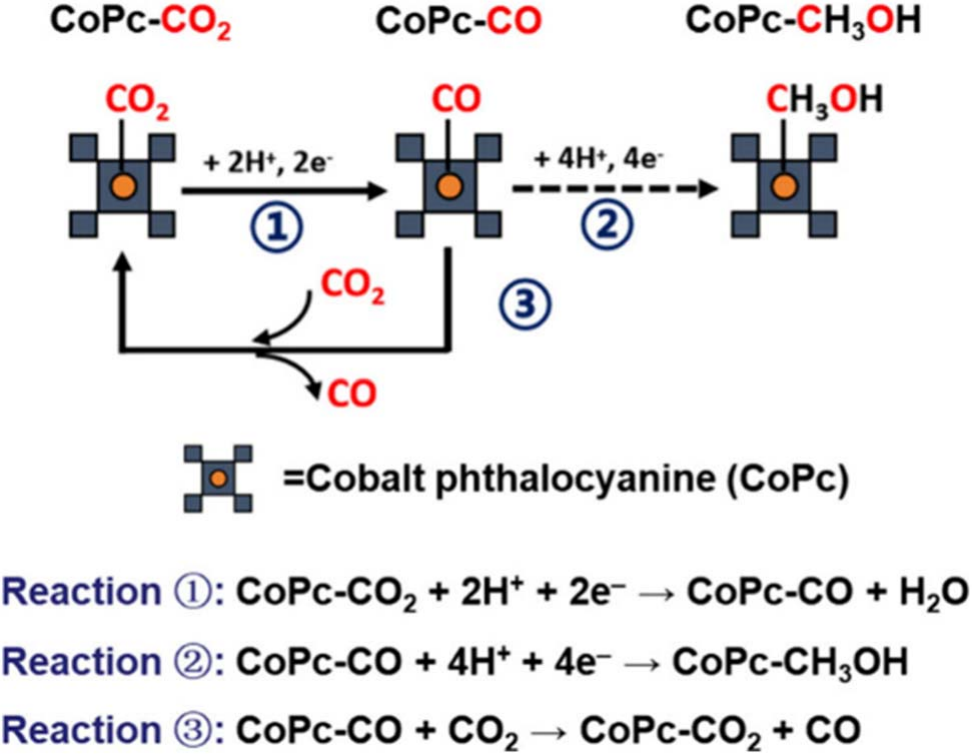
Schematic illustration of the CO_2_/CO competitive binding mechanism on CoPc. Reaction (1) represents the reduction from CO_2_ to CO. Reaction (2) is the further reduction from CO to CH_3_OH. Reaction (3) is the competitive displacement of CO by CO_2_.^73^ Reproduced from ref. 73 with permission from American Chemical Society, copyright 2024.

The mechanism of CH_3_OH formation in CO_2_ electroreduction has attracted intensive attention since the research of Boutin *et al.* and Wu *et al.* which showed that cobalt phthalocyanine anchored on multiwalled carbon nanotubes (CoPc/MWCNT) catalyzes CH_3_OH production *via* a CO_2_–CO–CH_3_OH cascade reaction in aqueous media, with CO serving as the key intermediate.^64,72^ As depicted in Fig. 4, the competitive binding dynamics between CO_2_ and CO intermediates on the CoPc active sites critically govern CH_3_OH selectivity.^73^ In this pathway, CO_2_ absorbs onto CoPc and is reduced to a CO-bound intermediate (Reaction (1)). Due to the weak CoPc–CO interaction, incoming CO_2_ readily displaces the adsorbed CO (Reaction (3)), favoring further CO_2_-to-CO conversion rather than CO-to-CH_3_OH progression (Reaction (2)).

According to the Sabatier principle, optimal CO binding strength to CoPc is essential to enable efficient CH_3_OH synthesis.^74–77^ However, experimental and theoretical studies quantified by Yao *et al.* through kinetic modeling reveal that CO_2_ exhibits a binding affinity to CoPc over three times stronger than that of CO, allowing CO_2_ to dominate active site occupancy.^77^ This competitive displacement explains the prevalence of CoPc and other metal phthalocyanines as CO catalysts with high selectivity. On the other hand, directly promoting the CO reduction reaction (CORR) to improve CH_3_OH yields remains challenging, as weak CO binding in CO-rich environments favors the HER over CH_3_OH production.^78,79^

**Fig. 5:**
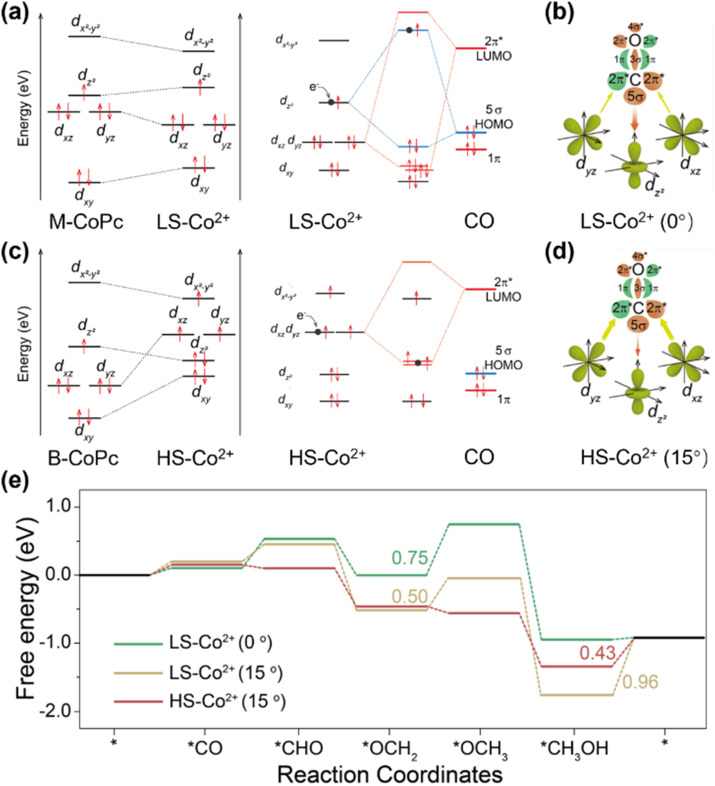
(a) and (c) 3d orbital diagrams of M-CoPc and B-CoPc after heat treatments and the corresponding illustration of interactions with CO molecular frontier orbitals (5σ and 2π*). Black dots represent the electrons from electrodes under cathodic bias. (b) and (d) Schematic of σ and π-donation bonds between CO and 3d orbitals of LS-Co^2+^ and HS-Co^2+^. (e) Free energy diagram of the CORR over LS-Co^2+^ and HS-Co^2+^.^74^ Reproduced from ref. 74 with permission from Springer Nature, copyright 2023.

Researchers focused on modifying the electronic properties of MPcs to promote the CO-binding affinity on the metal center and thus improve the CH_3_OH reduction performance. Ding *et al.* discovered that binuclear cobalt phthalocyanine (B-CoPc) undergoes a spin-state transition from low spin (LS, *S* = 1/2) to high spin (HS, *S* = 3/2) in its Co^3+^ 3d orbitals when thermally treated at 400 °C (Fig. 5).^74^ This transition is correlated with a loss of square-planar symmetry in B-CoPc, a structural distortion of 15° on the N–Co–N angle linked to enhanced CO_2_RR activity compared to mononuclear CoPc (M-CoPc). Experimental and DFT analyses revealed that the HS state shifts the rate-determining step (RDS) of the CORR from *CO/*CH_*x*_O protonation to CH_3_OH desorption, reducing the RDS energy barrier from 0.75 eV to 0.43 eV. From the perspective of electron configuration, the HS Co^2+^ center in B-CoPc features two unpaired electrons in its d_*xz*_/d_*yz*_ orbitals, which strengthen π back-donation to adsorbed CO. This electronic interaction strengthens the Co–C bond while weakening the C–O bond. The stabilized CO intermediate and destabilized C–O bond synergistically accelerate subsequent *CO hydrogenation steps, ultimately boosting CH_3_OH production. Consequently, the high-spin Co(ii) achieves significantly improved methanol selectivity and activity *via* the CORR compared to its low-spin counterpart.

**Fig. 6:**
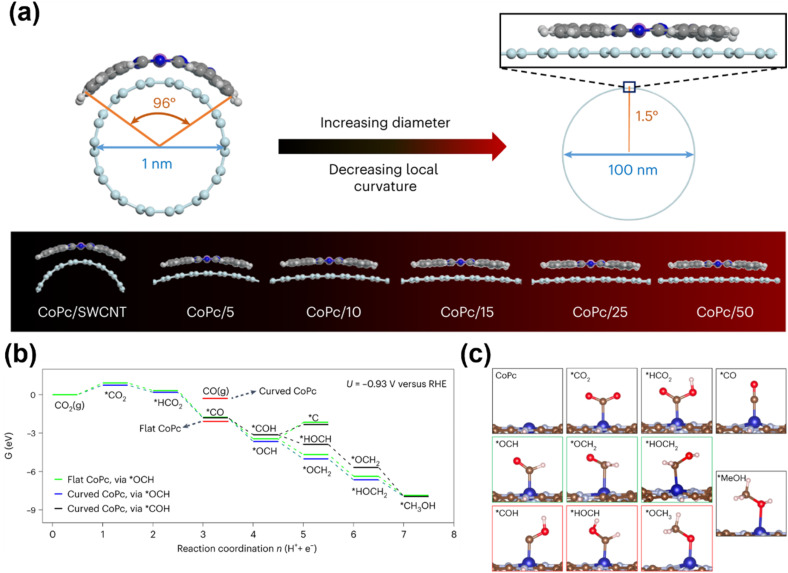
(a) Illustration of the structural distortion of CoPc on different-diameter CNTs, assuming that CoPc is fully elastic. (b) Free energies (eV) at *U* = −0.93 V *versus* RHE and pH 7 for CO_2_ reduction to methanol on curved and flat CoPc. Δ*G*_C_ and Δ*G*_F_ are reaction step free energies for the curved and flat CoPc, respectively. (c) The optimized intermediates of the CO_2_RR towards methanol on CoPc. Green boxes indicate the preferred intermediates, and red boxes indicate the higher-energy intermediates.^80^ Reproduced from ref. 80 with permission from Springer Nature, copyright 2023.

Enhancing CH_3_OH production at metal centers can be achieved through axial modification strategies as well, which tailor the electronic or geometric properties of active sites *via* axial ligand coordination or engineered catalyst–support interactions. Su *et al.* systematically investigated how CNT supports influence CO_2_-to-CH_3_OH conversion efficiency (Fig. 6).^80^ Their study revealed that single-walled CNTs (SWCNTs) with smaller diameters (∼1 nm) induce greater curvature in CoPc molecules compared to multi-walled CNTs (MWCNTs), owing to stronger interfacial interactions between CoPc and SWCNTs. This curvature reduces the N–Co–N bond angle within the CoPc macrocycle. The distorted, curved geometry of CoPc on SWCNTs strengthens CO adsorption across a range of electrochemical potentials relative to planar CoPc configurations. This enhanced CO binding facilitates subsequent hydrogenation steps critical for CH_3_OH formation. The work demonstrates how axial structural modulation can optimize intermediate binding energetics to boost methanol selectivity and activity, providing a blueprint for designing next-generation electrocatalysts through strategic catalyst–support engineering.

### 2.3 N_2_RR/NO_*x*_RR

**Fig. 7:**
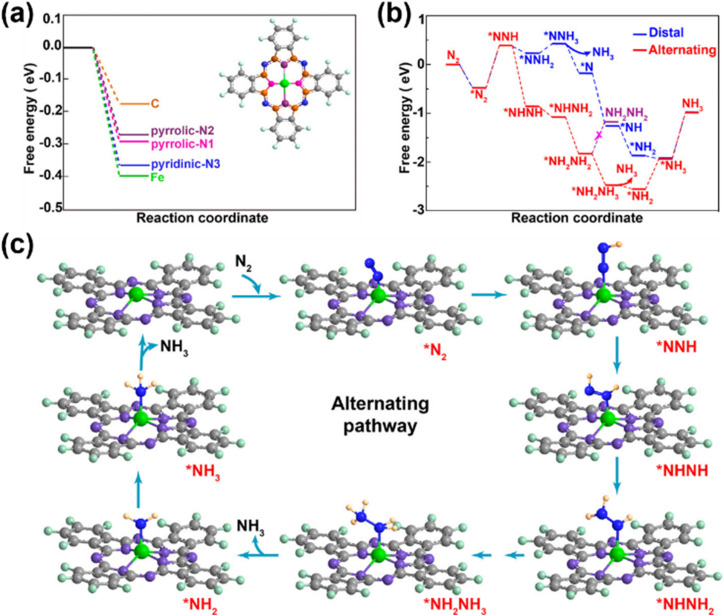
(a) Free-energy diagrams of N_2_ adsorption on five active sites of FePc, showing that the Fe site possesses the lowest energy barrier. (b) Different free-energy diagrams for the NRR on an Fe atom through distal and alternating mechanisms at zero potential. (c) Geometric structures of intermediates along the alternating pathway of the NRR proceeded on FePc.^82^ Reproduced from ref. 82 with permission from American Chemical Society, copyright 2019.

In 1989, Furuya and Yoshiba reported that MPcs immobilized on gas diffusion electrodes could catalyze the electrochemical reduction of nitrogen (N_2_) to ammonia (NH_3_) in aqueous media.^81^ Their work highlighted that the current efficiency and stability of phthalocyanine-based catalysts for the N_2_RR are strongly influenced by the identity of the central metal ion. Owing to its catalytic potential, FePc has become a focal point in N_2_RR studies. In 2019, He *et al.* developed a hybrid catalyst comprising FePc molecules dispersed on porous carbon (FePc/C), establishing it as a benchmark system for N_2_RR catalysis by MPcs (Fig. 7).^82^ Critical control experiments, including thiocyanate (SCN^−^) ion poisoning, confirmed that the N_2_RR takes place at the Fe center in FePc rather than on carbon or nitrogen moieties. Adsorption energy analyses further revealed that the Fe site exhibits the lowest energy barrier for N_2_ adsorption, favoring the spontaneous binding of N_2_ molecules. Mechanistic investigations into N_2_RR pathways demonstrated a preference for the alternating pathway (*via* *NNH → *NHNH intermediates) over the distal pathway (*NNH → *NNH_2_), as the latter involved higher free energy steps. The rate-determining step was identified as the initial *N_2_ → *NNH transition, with an energy barrier of 0.85 eV. Subsequent studies synthesized functionalized graphene sheets with finely dispersed FePc for the N_2_RR, revealing that π–π stacking interactions between FePc and graphene enhance catalytic activity by stabilizing the structure. Additionally, orbital rearrangement promotes end-on adsorption of N_2_ molecules at the Fe-N_4_ sites and stabilizes reaction intermediates, enabling selective NH_3_ production. To date, diverse metals (*e.g.*, Mn, Co, Ni, and Cu) coordinated within phthalocyanine frameworks have demonstrated N_2_RR potential, with improved catalytic performance through structural modifications to optimize N_2_ affinity and protonation efficiency.^83–86^

The electroreduction of a nitrite/nitrate ion (NO_2_^−^/NO_3_^−^) to NH_3_ (NO_*x*_RR) is a multi-step six- or eight-electron transfer process that generates many intermediates and by-products (*e.g.*, N_2_, NH_2_OH, N_2_H_4_, NO, and N_2_O). Compared to the N_2_RR, the NO_*x*_RR exhibits significantly faster reaction kinetics for NH_3_ synthesis, primarily due to the following circumstances: the high water solubility of NO_*x*_ species facilitates their accessibility to catalytically active sites; the dissociation energy of the N=O bond (204 kJ mol^−1^) is much lower compared to that of the N≡N triple bond (941 kJ mol^−1^); the higher theoretical redox potential of the NO_*x*_RR makes it thermodynamically more favorable than the N_2_RR. Furthermore, the NO_*x*_RR effectively mitigates interference from the competing HER, as the reduction potentials of the NO_*x*_RR and HER differ substantially. These advantages have induced research interest in the NO_*x*_RR for electrocatalytic NH_3_ production, with numerous studies exploring catalyst design and mechanistic pathways.

**Fig. 8:**
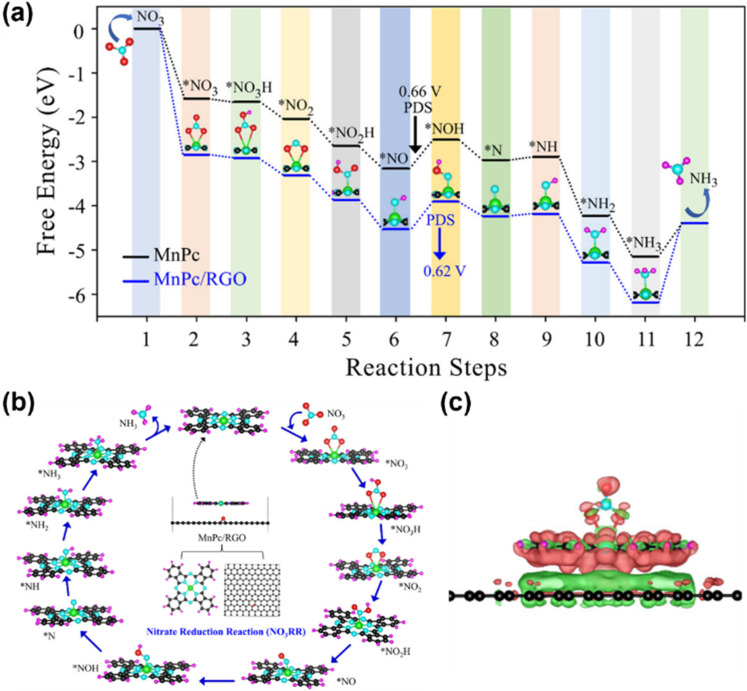
(a) Free energy profiles for both MnPc and MnPc/RGO systems; (b) reaction mechanism of the nitrate reduction reaction on the MnPc/RGO catalyst and the optimized structures of the intermediates. (c) Visualization of the charge density difference during NO_3_ adsorption on the MnPc/RGO catalyst.^88^ Reproduced from ref. 88 with permission from American Chemical Society, copyright 2023.

MPc has emerged as a type of highly efficient electrocatalyst for the NO_*x*_RR. Pioneering work by Chebotareva *et al.* demonstrated that MPcs (M = Fe, Co, Ni, Zn, Cu, Mn) immobilized on glassy carbon electrodes effectively catalyze NO_2_^−^/NO_3_^−^ reduction in alkaline media, with NH_3_ as the primary product.^87^ Among these, CuPc exhibited the lowest overpotential for the NO_*x*_RR. Subsequent studies revealed that dispersing MPcs on conductive carbon substrates (*e.g.*, carbon black and graphene) significantly enhances their electrocatalytic activity. For instance, Adalder *et al.* designed a heterostructure catalyst by anchoring MnPc on monolayer reduced graphene oxide (RGO), where strong π–π interactions between the graphene substrate and MnPc modulate the electron density of the Mn center, thereby optimizing NO_3_^−^ adsorption and activation (Fig. 8). The support reduced the energy barrier across all NO_*x*_RR steps, accelerating NH_3_ formation.^88^

Further mechanistic insights were provided by Harmon *et al.*, who investigated CNT-supported catalysts.^89^ They found that oxygen-containing groups on CNTs do not directly participate in the rate-determining step of NO_3_^−^ reduction but instead facilitate proton transfer, promoting competing HER and reducing NH_3_ selectivity. These findings indicate the importance of tailoring catalyst supports to suppress the HER while maintaining efficient charge/proton transfer. Collectively, these studies highlight how strategic modifications of MPc–support interactions can guide the design of high-performance NO_*x*_RR electrocatalysts.

## 3. Computation methods on MPc catalysts

### 3.1 Grand-canonical DFT (GC-DFT)

Most computational studies of electrochemical processes rely on conventional DFT, which assumes neutral, zero-potential conditions. These simulations typically incorporate solvation effects *via* implicit or explicit models while approximating applied potentials using the computational hydrogen electrode (CHE) framework.^90–92^ The CHE method adjusts DFT-derived energies of adsorbed species by using a term *neφ* (where *φ* is the applied potential and *n* is the formal electron count in the redox step), but it is limited to PCET intermediates and ignores critical interfacial effects of potential.^39,93^ Specifically, CHE neglects potential-induced electronic structure changes such as electron addition/removal, non-integer electron transfers, electric field polarization and structural relaxations of adsorbates at varying potentials. These simplifications under constant-charge assumptions often yield predictions that are different from experiments. For instance, CHE-based models consider CoPc SACs to be ineffective for the CO_2_RR and misjudge the priority for hydrogen evolution, yet experiments show high CO_2_RR activity with minimal hydrogen evolution of CoPc at −0.5 to −0.9 V *vs.* RHE.^20,94^ Similar discrepancies are observed for other CO_2_RR catalysts, underscoring the inadequacy of the CHE in modeling electrochemical interfaces.^95,96^

To address this, researchers have developed constant-potential simulation strategies. Nørskov proposed cell extrapolation and charge extrapolation to manually add different amounts of charge in the system to control the work function, but these methods require high calculation costs.^97–99^ A breakthrough emerged with grand canonical DFT (GC-DFT) developed by Sundararaman, which integrates joint density functional theory (JDFT)^100^ and continuum solvation models to explicitly simulate solid–liquid interfaces.^101^ GC-DFT self-consistently solves the Kohn–Sham equations while varying electron counts to maintain a fixed Fermi level, enabling direct modeling of potential-dependent reaction pathways. Though computationally intensive, when paired with advanced solvation models, GC-DFT captures potential-driven electronic and structural changes that are absent in conventional DFT, providing accurate explanations of experimental results. This method can reasonably describe the electrode–electrolyte interface and has been used to elucidate many electrochemical reactions in metal surfaces and SAC-based electrocatalysis.^102,103^

Brimley *et al.* utilized GC-DFT to analyze the influence of applied potential on the CO_2_RR energetics and electronic properties of 3d-block transition metal MN_4_C catalysts.^104^ Their calculations revealed a pronounced sensitivity of the *CO_2_ adsorption grand free energy to electrode potential across all MN_4_Cs, emphasizing the critical role of stabilizing the *CO_2_ intermediate to initiate the CO_2_RR. PDOS analysis further demonstrated nonlinear shifts in the metal d-orbital and CO_2_ electronic states at increasingly reductive potentials, showing how applied potential dynamically modulates the electrocatalyst electronic structure. Among the pyridinic MN_4_Cs studied, Sc-, Ti-, Co-, Cu-, and ZnN_4_C were identified as active for CO production at moderate to highly reducing potentials (−0.7 to −1.2 V *vs.* SHE). Notably, ZnN_4_C exhibited good CO_2_RR thermodynamics even at lower potentials, positioning it as a highly promising candidate for CO generation. Additionally, CoN_4_C displayed pH-independent CO_2_RR activity across all potentials, similar to molecular cobalt protoporphyrin catalysts. In contrast, Cr-, Mn-, and FeN_4_C suffered from prohibitively high CO desorption free energies, severely limiting their CO_2_RR rates. This study demonstrates the universality of the CO_2_RR mechanism in M–N–C type SACs and establishes a foundational framework for understanding MN_4_C electrocatalysts and provides critical theoretical guidelines for MPc catalyst design.

**Fig. 9:**
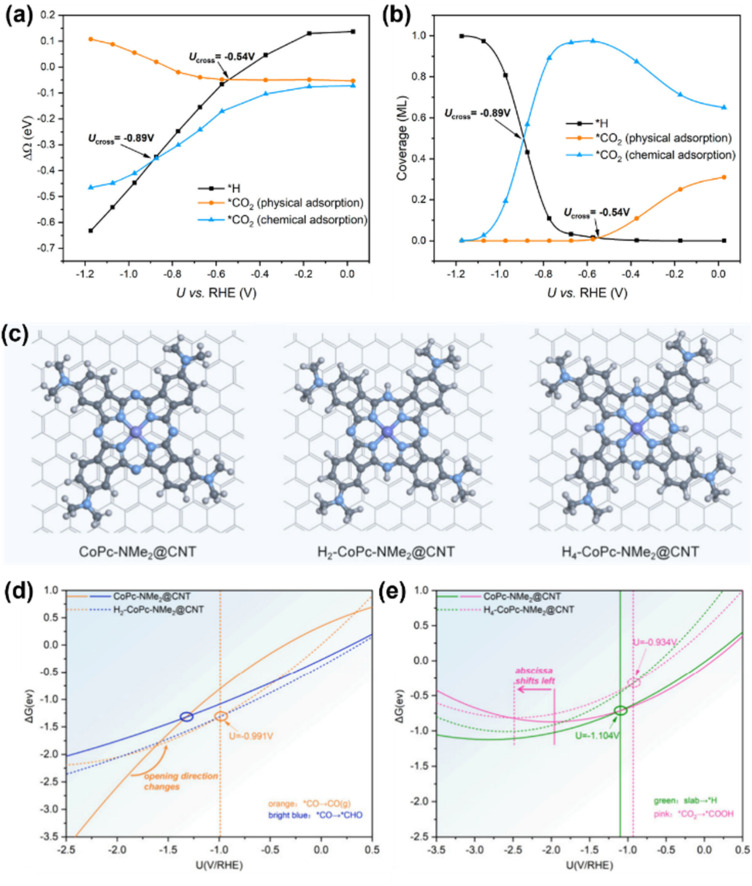
(a) Potential-dependent reaction energetics of physical and chemical adsorption of CO_2_ and *H formation on CoPc. (b) Potential-dependent competition of coverages among physical and chemical adsorption of CO_2_ and *H formation on CoPc.^105^ (c) Structural illustration of CoPc-NMe_2_@CNT, H_2_-CoPc-NMe_2_@CNT and H_4_-CoPc-NMe_2_@CNT. (d) and (e) Curves of Gibbs free energy change (Δ*G*) for the key steps determining the potential for maximum MeOH selectivity for CoPc-NMe_2_@CNT, H_2_-CoPc-NMe_2_@CNT and H_4_-CoPc-NMe_2_@CNT as a function of applied potential.^106^ Reproduced from ref. 105 and 106 with permission from American Chemical Society, copyright 2023, 2024.

To elaborate further, Wang *et al.* employed GC-DFT to unravel the interplay between the applied potential, CO_2_RR and hydrogen evolution on CoPc SACs.^105^ Their work systematically resolved how CO_2_ adsorption behavior evolves with applied potentials. Physical adsorption on CO_2_ dominates at low potential, while chemical adsorption prevails at higher potential due to enhanced surface charge, which activates CO_2_ by facilitating electron transfer. Besides, they revealed that protonation steps in the CO_2_RR exhibit distinct potential dependencies. The initial protonation (*CO_2_^−^ + H^+^ → *COOH) emerged as the potential-determining step with an onset potential of −0.46 V *vs.* RHE, while CO desorption showed negligible sensitivity to potential, as it involves minimal electron redistribution. Meanwhile, the competitive HER shared the same −0.46 V onset as the CO_2_RR. At a highly reducing potential, CO_2_RR activity decreases due to competitive surface coverage by adsorbed H and CO_2_ intermediates (Fig. 9(a) and (b)). These findings highlight the critical role of electron transfer dynamics in mediating intermediate coverage and catalytic selectivity under operational conditions.

Xu *et al.* developed dynamic product distribution on CoPc derivative catalysts based on the GC-DFT method.^106^ Their study identified CoPc functionalized with a strong electron-donating dimethylamino (–NMe_2_) group anchored on carbon nanotubes (CoPc-NMe_2_@CNT) as a promising catalyst that achieves high MeOH selectivity and good durability. Dynamic product distribution analysis revealed that the hydrogenated forms of the catalyst (H_2_-CoPc-NMe_2_@CNT and H_4_-CoPc-NMe_2_@CNT) maintain high MeOH selectivity, driven by a –NMe_2_ induced potential shift that optimizes intermediate stabilization. Besides, HER suppression was attributed to ligand hydrogenation, which weakens charge transfer from the cobalt center to adsorbed hydrogen atoms, thereby hindering H adsorption (Fig. 9(c–e)). This mechanism reveals the critical role of substituent-driven electronic modulation in balancing selectivity and stability. By correlating substituent effects with reaction dynamics, this work highlights the promise of strong electron-donating groups in CO_2_ electroreduction, offering important insights for advanced heterogeneous CoPc-based systems.

### 3.2 *Ab initio* molecular dynamics (AIMD)

AIMD was first developed by Car and Parrinello,^107^ and is used to model and understand the dynamic behaviors of atoms and electrons at the electrode–electrolyte interface under realistic conditions, providing mechanistic insights for catalyst design. Since AIMD is fundamentally limited by computational cost and timescale, a range of statistical and enhanced sampling methods have been developed to efficiently explore rare events and compute free energy surfaces, which are crucial for understanding reaction mechanisms.

Among these approaches, thermodynamic integration (TI) is a rigorous approach for determining free energy differences between states by integrating the ensemble-averaged derivative of the Hamiltonian along a coupling parameter (*λ*).^108^ These simulations are typically conducted with temperature control provided by the Nosé–Hoover thermostat, which maintains the system at constant temperature and ensures sampling within the canonical ensemble.^109,110^ The slow-growth method represents a typical case of TI, in which *λ* is incrementally varied during a single trajectory, with the accumulated work providing an estimate of the free energy difference.^111^ For those reaction processes described by a specific reaction coordinate, blue-moon sampling constrains the system along this coordinate, employing algorithms such as SHAKE to maintain the constraint, yielding accurate potential of mean force (PMF) profiles.^112,113^ Umbrella sampling^114^ and metadynamics^115^ are further enhanced sampling strategies: umbrella sampling applies biasing potentials to allow sampling of rarely visited regions along a known reaction coordinate, while metadynamics adds history-dependent bias to collective variables (CVs) to escape local minima and reconstruct multidimensional free energy landscapes. The selection of a suitable method depends on the system, the reaction pathway of interest, and the nature of the process. These methodologies, when combined with AIMD, enable detailed exploration of reaction mechanisms and energetics at electrode–electrolyte interfaces, providing both atomic-level insight and quantitative predictions that guide experimental catalyst development (Table 2).

**Table 2:** Comparison of AIMD-based free energy and enhanced sampling methods

Method	Purpose	Key feature	Limitation
Thermo-dynamic integration	Free energy difference	Integrates along *λ*	Requires multiple simulations
Slow-growth	Free energy difference	Gradual change in *λ* within one trajectory	Sensitive to the rate and may not be reversible
Blue-moon sampling	Free energy profile	Constrains a reaction coordinate	Requires an effective constraint algorithm
Umbrella sampling	Free energy profile	Applies biasing along the coordinate	Requires overlap between windows
Meta-dynamics	Free energy surface	Adds history-dependent bias to CVs	Depends critically on the choice of variables

In the early years, AIMD was applied for analyzing the CO_2_RR performance of cobalt porphyrin in water, integrated with DFT calculations with hybrid functionals and dielectric continuum solvation models.^116^ This advanced method has been used in the investigations of CoPc, which shows a better catalytic performance in CH_3_OH formation and possible C_2_ product formation.

**Fig. 10:**
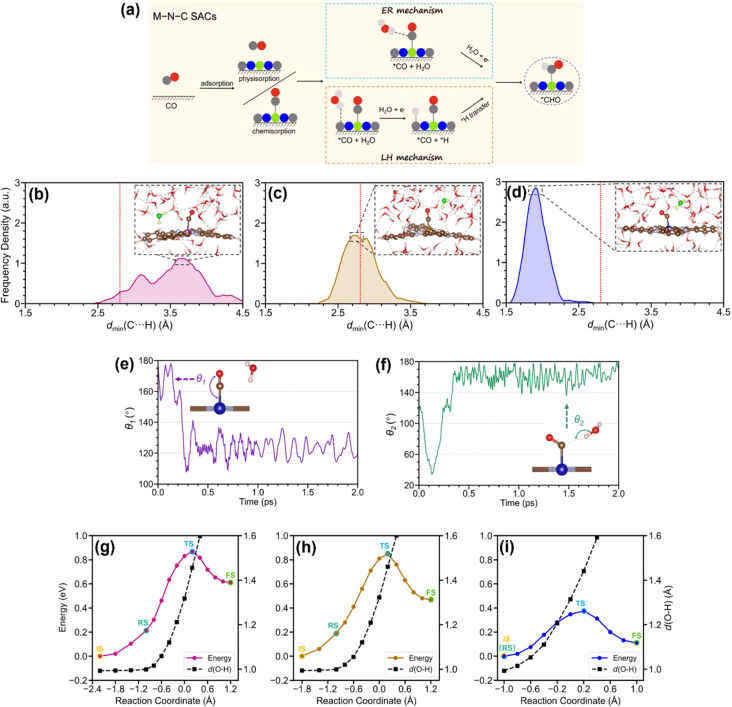
(a) Schematic diagram of the mechanistic pathways of CO reduction to *CHO *via* the ER and LH mechanisms on M–N–C SACs.^117^ (b)–(d) Frequency density distribution of *d*_min_(C⋯H) for (b) [MnPc]^2−^, (c) [FePc]^2−^, and (d) [CoPc]^2−^ in explicit water, respectively. The red dashed lines indicate a *d*_min_(C⋯H) of 2.84 Å. Representative snapshots from the trajectory are presented, showing instances in which *d*_min_(C⋯H) aligns with the peak of the frequency density. The H_2_O closest to *CO is highlighted. (e) and (f) The evolution of the Co–C–O angle (*θ*_1_) and the C⋯H–O angle (*θ*_2_) during the first 2 ps of the molecular dynamics simulation. Snapshots of the Co atom with *CO and the closest H_2_O are included, corresponding to the simulation time indicated by the arrowheads. (g)–(i) Free energy profile and evolution of average *d*(O–H) along the reaction coordinate for (g) FePc, (h) MnPc, and (i) CoPc systems. The reaction coordinate is quantified by the collective variable of *ξ* = *d*(O–H) − *d*(C–H).^119^ Reproduced from ref. 117 and 119 with permission from American Chemical Society, copyright 2023, 2024.

Liu *et al.* employed AIMD simulations to highlight the key role of intermolecular interactions in driving the Eley–Rideal (ER) protonation and Langmuir–Hinshelwood (LH) protonation of *CO during CO hydrogenation on metal–nitrogen–carbon (M–N–C) SACs (Fig. 10(a)), which was on the explicit water bilayer and lasted for 10 ps while maintaining a temperature of 300 K based on the Nosé–Hoover thermostat.^117^ In the ER pathway, adsorbed *CO directly accepts hydrogen from nearby water molecules, whereas the LH mechanism involves a two-step process with an adsorbed *H species (formed *via* water dissociation) that couples with *CO at separate active sites.^118^ Further analysis by Li *et al.* revealed a unique C⋯H–O hydrogen bond, formed exclusively between water and *CO on charged CoPc, where an antibonding electron pair acted as a proton acceptor.^119^ They adapted a combination of slow-growth and blue-moon sampling methods with the SHAKE algorithm for the free energy profiles of *CO protonation. Unlike MnPc and FePc systems, the proton transfer to *CO does not involve the diffusion of the water molecule, and the C⋯H–O hydrogen bond additionally reduces the energy barrier for O–H bond dissociation and C–H bond formation. This unique interaction significantly accelerated CO protonation *via* the ER pathway, explaining the enhanced multielectron CO_2_RR activity of CoPc. They highlighted the electronic structure of CoPc as critical to this hydrogen bond formation: the half-filled d_*z*^2^_ orbital of the Co ion enabled CO chemisorption, while orbital alignment between CoPc and CO facilitated occupation of the Co–CO σ antibonding orbital at operational potentials (Fig. 10(b–i)). Their work demonstrates hydrogen-bonding interactions that optimize proton transfer in electrochemical systems, offering a strategic route to enhance catalytic efficiency.

Li and his colleagues investigated the free-energy profiles of the CO_2_RR on CoPc with graphene at varying electrode potentials using constrained AIMD simulations combined with TI and fully explicit solvation models, which were sampled by the canonical ensemble employing the Nosé–Hoover thermostat with a time step of 1.0 fs at the target temperature of 300 K.^120^ Their study revealed that the reaction free energy, energy barrier, and transition-state location during CO_2_ adsorption are strongly influenced by the applied electrode potential. Furthermore, they identified the proton-transfer step as the rate-determining process for the CO_2_RR on CoPc, elucidating its pH-dependent behavior. The energy barrier of this critical step could be effectively lowered by applying more negative potentials or introducing electron-withdrawing substituents to the phthalocyanine framework, providing mechanistic insights into tailoring catalytic performance through electronic and electrochemical modulation.

### 3.3 Machine learning (ML)

As a subset of artificial intelligence, machine learning (ML) enables systems to learn from data, identify patterns, and make decisions with minimal human intervention by building analytical models automatically.^121^ Compared to traditional DFT calculations, ML methods effectively reduce computational costs while achieving high predictive accuracy when paired with appropriate algorithms and feature engineering. This efficiency has promoted the innovative integration of DFT and ML into a hybrid computational framework, combining the accuracy of DFT with the speed of ML by training ML models on DFT-calculated data. In this approach, DFT is used to generate a dataset of key properties, such as total energies, atomic forces, or electronegativities, which are then paired with carefully chosen descriptors of the atomic structure to train an ML model. The primary goal of the ML component is to learn the mapping from structure to property, enabling rapid and accurate predictions of DFT-level properties for new, unknown structures without the need for further costly quantum mechanical calculations. This combined approach nowadays has widely been applied in catalysis research, such as active oxygen evolution catalyst screening, the CO_2_ reduction network on crystalline solids, and SAC exploration, which allows for the prediction of catalytic performance in complex systems and uncovers intrinsic descriptors governing their underlying activity.^122–124^ To assess the accuracy of ML models, two metrics—root-mean-square error (RMSE) and the coefficient of determination (*R*^2^ score)—are commonly employed. The *R*^2^ score, ranging from 0 to 1, reflects prediction quality, where values closer to 1 indicate good performance. RMSE quantifies the deviation between predicted and actual values, where lower values represent better model performance.

**Fig. 11:**
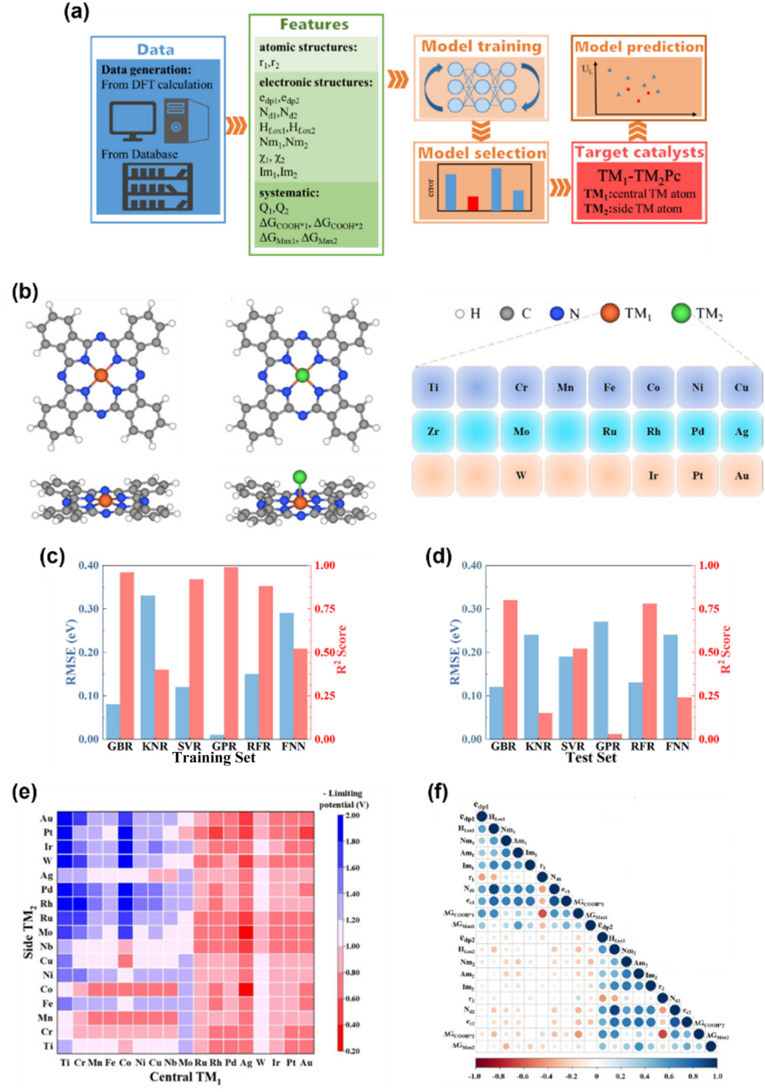
(a) Schematic diagrams of the procedure of the machine-learning-accelerated catalytic activity prediction of Pc DMSCs, and the selected features are listed in the green box. (b) Structures of TMPc and TM_1_-TM_2_Pc, respectively, together with all screened transition metal elements. (c) and (d) Comparison of the RMSE and the *R*^2^ score for each model on the training set and the test set. (e) The heat map of ML-predicted theoretical limiting potentials of Pc DMSCs. The redder the color, the better the catalytic activity for the CO_2_RR. (f) Pearson correlation map between each of the 20 input features. Red and blue colors correspond to strong and direct correlation, while white represents no correlation.^139^ Reproduced from ref. 139 with permission from American Chemical Society, copyright 2021.

In recent years, researchers have employed ML algorithms integrated with DFT methods to screen MPcs for electrocatalysis by predicting the catalytic performance and revealing intrinsic descriptors of the catalytic systems.^125,126^ Wan *et al.* developed a DFT-based ML method for catalysis program (DMCP) based on ten common algorithms to implement the DFT-ML scheme.^127^ These ML models can be grouped into several algorithmic families. Tree-based ensemble methods include gradient boosted regression (GBR),^128,129^ random forest regression (RFR),^130^ and extra trees regression (ETR).^131^ All these build collections of decision trees either sequentially, in parallel, or with added randomness to enhance predictive accuracy and robustness. Linear and regularized regression techniques such as kernel ridge regression (KRR),^132^ least absolute shrinkage and selection operator (LASSO),^133^ and elastic net regression (ENR)^134^ extend linear regression by introducing regularization (L1, L2, or both) or kernel methods to capture nonlinearities and select relevant features. Instance-based learning is represented by k-nearest neighbor regression (KNN),^135^ which bases predictions on the similarity between new samples and known data points. Kernel-based and probabilistic models, including support vector regression (SVR)^136^ and Gaussian process regression (GPR),^137^ use kernels to handle nonlinear patterns, with GPR also providing uncertainty estimates for its predictions. Finally, feedforward neural networks (FNNs)^138^ are a type of deep learning model that uses layered, interconnected nodes to capture complex, nonlinear relationships in data. Using DMCP to investigate the CO_2_RR on phthalocyanine dual-metal-site catalysts (Pc DMSCs), they applied an 8 : 2 ratio for the training and test sets through random shuffling and division. The results demonstrated that the GBR model is the best-performing and most well-trained machine learning model, achieving high accuracy with an RMSE of 0.08 eV and an *R*^2^ score of 0.96.^139^ Among 289 Pc DMSC candidates, Ag-MoPc emerged as a highly stable and efficient electrocatalyst, exhibiting a low limiting potential of −0.33 V, and the ML prediction error is only 0.02 V. The DFT-ML hybrid approach proved approximately sevenfold faster than conventional DFT methods (Fig. 11). This methodology bridges computational efficiency and precision, offering a scalable strategy to identify optimal catalytic materials for the CO_2_RR while drastically reducing resource demands.

Zhang and Wang investigated the CO_2_RR to CO on NiPc using a combined DFT and ML approach.^140^ Their DFT calculations revealed that the adsorption properties and structural stability of the Ni–N_4_ active site depend on the electron density around the Ni center, which can be modulated by attaching electron-withdrawing or electron-donating groups. They also explored the interaction between NiPc and nanocarbon substrates, uncovering a correlation between the π–π stacking interaction energy and charge transfer. Notably, this interaction strength was governed by the polarizability and chemical nature of the substituent rather than electronic effects on the Ni–N_4_ moiety, suggesting a novel guidance for molecular design. On the other hand, they developed a descriptor-driven ML framework, employing genetic algorithms, semiempirical quantum calculations, and deep neural networks to screen substituted NiPc derivatives. This computational pipeline identified several high-performing candidates, with the optimal molecule surpassing the leading reference catalyst by 100 mV in reduction potential while maintaining good stability. The study provides a general blueprint for designing hybrid catalytic materials, balancing electronic modification and interfacial interactions to optimize performance across diverse applications.

ML has been extended to optimize MPc catalytic systems apart from CO_2_ reduction. Wang and collaborators systematically assessed 224 carbon-supported SACs, including MPcs, for the HER and OER.^141^ Their analysis identified CoPc as a HER catalyst candidate, achieving an overpotential below 0.15 V, while Co/Rh/Ir-Pc SACs emerged as competitive OER alternatives to conventional IrO_2_. Furthermore, CoPc exhibited bifunctional activity and stability for overall water splitting. To decode these trends, they trained the GBR model and highlighted that HER activity in SACs is predominantly determined by the spatial arrangement of atoms around the transition metal site and the electronic characteristics of the metal. This approach not only accelerates catalyst discovery but also provides atomistic insights into tailoring active sites for renewable energy applications. Xia *et al.* used the RFR method to analyze the influence of physicochemical properties on ORR performances for the carbon materials and discovered the two-dimensional defective nitrogen-doped graphene nanomesh (NGM) as a promising candidate for loading catalysts.^142^ They successfully synthesized the FePc/NGM catalyst and examined the ORR performance, showing the potential of bridging the ML and synthetic chemistry for the discovery of novel catalysts for practical applications.

## 4. Summary and outlook

Metal phthalocyanines (MPcs) have emerged as versatile molecular catalysts for electrochemical energy conversion, offering tunable electronic structures, well-defined active sites, and adaptability across diverse reactions such as the oxygen reduction reaction (ORR), oxygen evolution reaction (OER), hydrogen evolution reaction (HER), CO_2_ reduction reaction (CO_2_RR), and nitrogen/nitrate reduction reaction (N_2_RR/NO_*x*_RR). The integration of MPcs with carbon supports such as graphene and carbon nanotubes significantly enhances their catalytic performance. From a mechanistic point of view, these carbon substrates provide a high surface area and electrical conductivity, facilitating efficient electron transfer during catalysis. Additionally, strong π–π interactions between MPcs and the carbon matrix promote uniform dispersion of active sites and prevent aggregation, thereby increasing the number of accessible catalytic centers. Moreover, the carbon-based supports also induce charge redistribution and structural modulation at the interface, which optimizes the adsorption of reaction intermediates and improves reaction kinetics. In general, these synergistic effects contribute to enhanced activity, selectivity, and stability of MPc-based catalysts in various electrochemical reactions. Advances in computational methods, such as grand-canonical DFT (GC-DFT) and *ab initio* molecular dynamics (AIMD), have elucidated the mechanistic details of MPc-catalyzed processes, revealing potential-dependent pathways, solvent effects, and interfacial dynamics. Machine learning (ML) further accelerates catalyst discovery by bridging computational efficiency with predictive accuracy, enabling rapid screening of substituents, metal centers, and substrate interactions to tailor catalytic selectivity and activity.

Looking ahead, the field faces challenges in translating theoretical insights into practical applications. Future research should mainly focus on enhancing the durability and scalability of MPc-based catalysts under operational conditions. Up to now, the most recently reported dimethylamino group-decorated NiPc catalysts for CO production reached nearly 100% CO_2_-to-CO reduction selectivity, exhibiting long-term stability by preserving more than 98% CO selectivity for over 40 hours at a current density of 100 mA cm^−2^.^143^ However, both the current density and the duration of stable operation remain below the thresholds required for practical commercial application. Besides, MPc catalysts need to address competitive side reactions like the HER through advanced electronic and geometric engineering. Moreover, integrating multi-scale computational frameworks—combining DFT, AIMD, and ML—with experimental validation will refine predictive models and uncover novel descriptors for catalytic performance. The AI-driven modulation and screening based on the descriptors provides efficient investigations with low computational cost, offering the chance to unravel unknown mechanisms in a more complicated situation. Apart from the DFT + ML hybrid approach, the AIMD + ML hybrid approach, considering the potential or free energy change, is also considered to be a promising strategy to supply accurate, scalable, and cost-effective simulations in the future, expanding the scope of molecular and materials modelling to more complex systems and longer time scales. Such integrations are able to alleviate the gap between theoretical calculations and practical experiments, which offer more significant insights into the reaction processes and accelerates the advances of current catalyst designs.^144^ The future DFT and AIMD techniques intersected by ML in mechanism studies and environment simulations will enable the application of MPcs in practical applications.

## Author contributions

Z. L. performed the literature search, analyzed the published results, and wrote the manuscript. Z. Z., M. S., T. W., Q. L., L. L., B. C., C. H. C., and H. H. W. assisted with the writing of the manuscript. B. Huang provided key advice and supervised the preparation of the review.

## Conflicts of interest

There are no conflicts to declare.

## Data Availability

No primary research results, software, or code have been included, and no new data were generated or analysed as part of this review.
